# Grand Challenges for Medtech Data Analytics

**DOI:** 10.3389/fmedt.2019.00002

**Published:** 2019-12-17

**Authors:** Yu-Dong Zhang, Qinghua Zhou

**Affiliations:** School of Informatics, University of Leicester, Leicester, United Kingdom

**Keywords:** Medtech data analytics, artificial intelligence, deep learning, data-driven model, big data

The capacity and affordability of data storage rapidly increased over the past century (1). In the twentieth century, punch cards were widely used by controlling a loom to punch holes in a paper tape. In the 1960s, the magnetic storage replaced the punch cards. In 1947, a practical random-access memory (RAM) was invented in the form of “Williams tube.” The earliest RAM can store 1,024 bits data. IBM sold the first floppy disk in 1971. The most common 51/4-inch diskette allows a 360 KB storage. Optical disks come to the use by Sony and Phillips in mid-1980s. Its first version has a capacity of 650 MB data, while the latest Blu-ray disc can hold 25 GB storage. We also have flash drives nowadays for portable storage, with capacity varying from 8 to 128 GB. In the field of persistence storage, the hard disk drive (HDD) is dominant. The typical capacity of HDD varies from 60 GB to 8 TB. Another device is called solid-state drives (SSD), which does not use the conventional spinning disks and movable heads. SSDs store data in semiconductor cells, and are more resistant to physical shock. All those innovations are the basis of our current data abundant society.

The appearance of data silos and cloud computing storage all facilitate the use of big data. Data silos are insular data repositories which work under the control of one organization and is isolated from the rest. If data silos are plant-specific electric generators, then cloud computing is the electricity grid. Cloud computing facilitates the provision of on-demand resources and helps utilize the data more efficiently. All those techniques lay a solid foundation for the popularity of big medical data (2).

The first grand challenge is the “heterogenous” data. The available medical data to us today are a mixture of structured, semi-structured, and unstructured data (3). Data sources include medical imaging, genomic sequencing, patient engagement platforms, e-health records, mobile-phone apps, health-care social media, monitoring, and wearable devices, etc. One possible solution to this heterogeneity in data analysis is to use “*data fusion*” methods (4), Data fusion integrates the heterogeneous data in attempts to create better-performing analytical models compared to models using data of single modality.

The second grand challenge is how to handle the “big” medical data. International Data Corporation (IDC) once predicted that “the global datasphere will grow from 33 zettabytes in 2018 to 175 zettabytes by 2025” (5). Recall that 1 zettabyte is 10 to the power of 21 bytes (6). Those vast amounts of mixture data bring challenges: the lack of data standardization, the concern of privacy and security issues, the speed-limitation and eavesdrop possibility of data transfer, the reliability of data storage, etc. These challenges have slowed the process of leveraging healthcare data and deployment of existing analytics models. High-speed computer servers with the integration of high distributed computing, streaming algorithms, or cloud computing (7) are possible solutions to this challenge.

The third grand challenge is to generalize a clear “definition” of MedTech data analytics, an interdisciplinary field that builds upon big data, health data analysis, data-driven model, artificial intelligence, etc. A clear definition is necessary so that the users can know what MedTech is and is not, and how to approach it appropriately. Prof. Dan Ariely once said, “*Big data is like teenage sex: everyone talks about it, nobody really knows how to do it, everyone thinks everyone else is doing it, so everyone claims they are doing it…*”(8). This ambiguity of “big data” applies to AI, to deep learning, to almost all the emerging techniques that are currently being or will be applied to MedTech data analysis. A potential solution is to create an accurate and exact methodology framework so users can easily understand those concepts.

The fourth grand challenge is “*small-size*.” Although the medical data are often heterogeneous and substantial for a single subject, the size of the patient cohort is usually quite small compared to healthy controls. This commonly seen categorical imbalance, i.e., unbalanced datasets, will cause the so-called “*overfitting*” problem to not only classical AI models but also modern deep learning models. In training, models can become too closely related to the datasets' majority of healthy controls. Some remedies were proposed to avoid overfitting, such as cost matrix, early stopping, oversampling, sensitivity analysis (9), etc.

The fifth grand challenge is the “*reproducibility crisis*” (10). Currently, hold-out and k-fold cross-validation (11) are the commonly accepted methods by statisticians working on medical data. However, in practice, a slight change on the hyperparameters (e.g., the value of hold-out ratio or number of folds) can lead to different performance results. Authors even reported inconsistent results to published literature using the same dataset and the same configuration. Hence, it is desired to have more reliable validation techniques that go beyond the current statistical validation techniques. We may need to use more robust experimental designs, better mentorship, and more reliable statistics.

The sixth challenge is the “*privacy*” and “*ownership*” problem. We need to maintain the confidentiality of patients' records from their employers, insurance company, and society. Current electronic health records (EHR) (12) and patient care management systems (PCMS) can protect medical information to some degree. However, there are significant public concerns in the lack of strategies to deal with privacy threats such as nature/environment, hackers, technology failures, etc. Furthermore, the development of new artificial intelligence techniques may increase the threat to privacy. For example, recent research have shown advanced facial AI reconstruction techniques can reconstruct facial appearance from MRI images (13). The General Data Protection Regulation (GDPR) help and regulate scientists and technicians in the protection of medical data privacy while also emphasizing the shortcomings of current health data management. More strict laws are expected to take effect by legislatures, and more reliable encryption methods are needed by IT technicians to help protect the privacy of medical data.

Although we come across the challenges as stated, MedTech data analysis is going through a rapid change every passing day. The section “Medtech Data Analytics” is part of the journal “Frontiers in Medical Technology.” Our goal is to help solve the above challenges. The orientation of this section is toward papers that facilitate the generation of data-driven models for medical data. This section will highlight leveraging emerging techniques to help explore analytics in big medical data applications, with welcome to traditional signal processing techniques and novel artificial intelligence methods are welcomed. The techniques and methods of interest include: data mining, artificial intelligence, machine learning, deep learning, knowledge discovery, predictive analysis from medical data, disease diagnostic data-driven models, healthcare workflow mining, hospital readmission and patient length of stay analytics, medical IoT and sensor data quality and reliability, disease profiling and personalized medicine, healthcare cost/service modeling, social media and cloud-computing based analytics for public health, medical expert system and decision support system, natural language processing and text mining, generating medical imaging labels, evidence-based recommender systems, clinical phenotyping, surgery planning, and real-time visualization techniques for the query and analysis of medical data.

Furthermore, Medtech Data Analytics aims to find new biomarkers, improve our understanding of disease mechanisms, increase the efficiency in healthcare delivery, reduce the overall cost for patient/family/hospital, and facilitate clinical decision support. This section encourages submissions of scientific or technical findings from both academia and healthcare industry to accelerate the addressing of all these challenges.

We have made an influential start, especially by inviting a remarkable team of world-famous associate editors, and by leveraging on the abundant resources of the new coming Frontiers in Medical Technology of the leading Frontiers academic press. We look forward to seeing how this journal and this section will grow.

## Author Contributions

Y-DZ designed and wrote the draft. QZ organized references, gave critical comments, and revised English.

### Conflict of Interest

The authors declare that the research was conducted in the absence of any commercial or financial relationships that could be construed as a potential conflict of interest.
